# Conversion of RpoS^−^ Attenuated *Salmonella enterica* Serovar Typhi Vaccine Strains to RpoS^+^ Improves Their Resistance to Host Defense Barriers

**DOI:** 10.1128/mSphere.00006-18

**Published:** 2018-02-28

**Authors:** Whittney N. Burda, Karen E. Brenneman, Amanda Gonzales, Roy Curtiss

**Affiliations:** a Department of Infectious Disease and Immunology, College of Veterinary Medicine, University of Florida, Gainesville, Florida, USA; b The Biodesign Institute and School of Life Sciences, Arizona State University, Tempe, Arizona, USA; University of Kentucky

**Keywords:** RASV, RpoS, *S*. Typhi vaccines, *Salmonella*, recombinant

## Abstract

The vast majority of live attenuated typhoid vaccines are constructed from the *Salmonella enterica* serovar Typhi strain Ty2, which is devoid of a functioning alternative sigma factor, RpoS, due to the presence of a frameshift mutation. RpoS is a specialized sigma factor that plays an important role in the general stress response of a number of Gram-negative organisms, including *Salmonella*. Previous studies have demonstrated that this sigma factor is necessary for survival following exposure to acid, hydrogen peroxide, nutrient-limiting conditions, and starvation. In addition, studies with *Salmonella enterica* serovar Typhimurium and the mouse model of typhoid fever have shown that RpoS is important in colonization and survival within the infected murine host. We converted 4 clinically studied candidate typhoid vaccine strains derived from Ty2 [CVD908*-htrA*, Ty800, and χ9639(pYA3493)] and the licensed live typhoid vaccine Ty21a (also derived from Ty2) to RpoS^+^ and compared their abilities to withstand environmental stresses that may be encountered within the host to those of the RpoS^−^ parent strains. The results of our study indicate that strains that contain a functional RpoS were better able to survive following stress and that they would be ideal for further development as safe, effective vaccines to prevent *S*. Typhi infections or as vectors in recombinant attenuated *Salmonella* vaccines (RASVs) designed to protect against other infectious disease agents in humans. The *S*. Typhi strains constructed and described here will be made freely available upon request, as will the suicide vector used to convert *rpoS* mutants to RpoS^+^.

**IMPORTANCE** Recombinant attenuated *Salmonella* vaccines (RASVs) represent a unique prevention strategy to combating infectious disease because they utilize the ability of *Salmonella* to invade and colonize deep effector lymphoid tissues and deliver hetero- and homologous derived antigens at the lowest immunizing dose. Our recent clinical trial in human volunteers indicated that an RpoS^+^ derivative of Ty2 was better at inducing immune responses than its RpoS^−^ counterpart. In this study, we demonstrate that a functional RpoS allele is beneficial for developing effective live attenuated vaccines against *S*. Typhi or in using *S*. Typhi as a recombinant attenuated vaccine vector to deliver other protective antigens.

## Abstract (precis)

Recombinant attenuated *Salmonella* vaccines (RASVs) represent a unique prevention strategy to combating infectious disease because they utilize the ability of *Salmonella* to invade and colonize deep effector lymphoid tissues and deliver hetero- and homologous derived antigens at the lowest immunizing dose. Our recent clinical trial in human volunteers indicated that an RpoS^+^ derivative of Ty2 was better at inducing immune responses than its RpoS^−^ counterpart. In this study, we demonstrate that a functional RpoS allele is beneficial for developing effective live attenuated vaccines against *S*. Typhi or in using *S*. Typhi as a recombinant attenuated vaccine vector to deliver other protective antigens.

## INTRODUCTION

Despite attempts at controlling and eliminating infectious diseases through the use of both prophylactic vaccination and antimicrobial therapy, they still remain a major public health concern for the world. Thus, every year approximately 300 million new infections and over 10 million deaths occur worldwide (1). Prophylactic vaccination is the most efficient and cost-effective way of preventing disease; however, development of proper vaccination and immunization strategies remains a challenge. Research suggests that the most effective vaccination strategy is through the use of live attenuated bacterial strains because they are more immunogenic and induce a longer-lasting immune response than inactivated vaccines (2, 3). However, balancing attenuation and immunogenicity can be difficult when developing a live attenuated vaccine.

For the last three decades, the Gram-negative pathogen *Salmonella enterica* has been used to develop live attenuated vaccine vectors to deliver homologous and heterologous antigens to the immune system (4–10). These recombinant attenuated *Salmonella* vaccines (RASVs) have been shown to induce both strong humoral and cellular immune responses to a variety of pathogenic bacteria, viruses, and even cancer cells (7, 8, 11–13). RASVs using attenuated *Salmonella enterica* serovar Typhi strains as recombinant vector strains for humans (RASTyVs) include Ty21a, Ty800, CVD908-*htrA*, and χ9639 (14–20). Ty21a is a currently licensed live attenuated vaccine in the United States and Europe that is recommended to persons traveling to countries where typhoid fever, which is caused by *S*. Typhi, is endemic (15). Ty21a was derived from the *S*. Typhi strain Ty2 through random mutagenic methods, which rendered it both *galE* and Vi negative (15). Another Ty2-derived vaccine is Ty800, which was made through deleting *phoPQ*, which encodes a two-component system that controls virulence gene expression (16, 21–25). Like Ty21a, Ty800 was developed to combat typhoid fever (16). However, unlike Ty21a, which requires three doses to achieve maximum protection, Ty800 requires only a single dose (16). CVD908-*htrA* was developed both as a potential vaccine against typhoid and as a vector to carry heterologous antigens (19, 20). Derived from the Ty2 parent strain, CVD908-*htrA* is also nutritionally auxotrophic for aromatic metabolites (i.e., *p*-aminobenzoic acid [PABA] and 2,3-dihydroxybenzoate [DHB]), which are not found within mammalian tissues, and the *htrA* gene, which encodes a heat shock protein that has been shown to be important in virulence (20, 21, 26, 27). The χ9639 strain, which was also derived from Ty2, was developed to deliver heterologous antigens (18). This strain was developed by the Curtiss laboratory and relies on arabinose-dependent regulated synthesis of virulence genes that results in regulated delayed attenuation and regulated delayed synthesis of recombinant protective antigens *in vivo* (18).

Previous work with the *S*. Typhi Ty2 strain demonstrated that it has a frameshift mutation in the major transcriptional regulator RpoS (28). This regulator, also known as the stationary-phase sigma factor, is known to control the expression of genes that play a critical role in survival of several stresses, including acid stress, oxidative stress, and starvation (29–32). By using *Salmonella enterica* serovar Typhimurium and the mouse model for typhoid fever, RpoS has been shown to enhance the ability to overcome innate host defenses, such as the acidity of the stomach, reactive oxygen radicals produced by immune cells, and nutrient-limiting conditions of the host tissue (33, 34). In addition, RpoS has been shown to be important in virulence and establishing *S*. Typhimurium infection, which is beneficial to vaccine strains in order to deliver antigens to mucosal immune cells (35–37). Studies have demonstrated that RpoS is critical for persisting in lymphoid organs such as the spleen and liver and for the initial stages of *S*. Typhimurium infection in murine Peyer’s patches (gut-associated lymphoid tissues [GALT]) (37–39). This leads us to believe that the presence of a functional RpoS in RASVs would enhance the survival of the strains as they make their way to the mucosa-associated lymphoid tissues.

Previous studies performed by our lab suggest that *S*. Typhi-derived RASV strains containing a functional RpoS (RpoS^+^) are also, like *S*. Typhimurium RpoS^+^ strains, superior to strains that are deficient in RpoS (RpoS^−^) in inducing immune responses (14, 18). Therefore, we constructed RpoS^+^ variants of the *S*. Typhi-derived Ty21a, Ty800, CVD908-*htrA*, and χ9639 RASV strains to evaluate their abilities compared to the parent strains to withstand different environmental and host defense stresses encountered after oral administration. These studies were predicated on the belief that this would enhance their efficiency in colonizing the GALT and internal effector lymphoid tissues when used to protect against typhoid fever or when used as vectors in RASVs to induce protective immunity against heterologous pathogens in humans. Since the parents of the newly constructed strains are either licensed or have demonstrated safety and efficacy in multiple phase I trials, we will make these strains freely available upon request in hopes of further enhancing the efficacy of these licensed and candidate *S*. Typhi vaccines and vaccine vectors. Full strain descriptions appear in Table 1.

**TABLE 1:** Strains, plasmids, and primers used in this study

Strain or plasmid	Genotype or description	Source, derivation, or reference(s)
Strains		
*E. coli* χ7213	K-12; *thi-1 thr-1 leuB6 fhuA21 lacY1 glnV44* Δ*asdA4 recA1* RP42-Tc::Mu [λpir] Km^r^	60
*S*. Typhi		
χ3769	Ty2; RpoS^−^	61
χ8073	CVD908-*htrA*; Δ*aroC1019* Δ*aroD1013* Δ*htrA*	15, 17
χ8205	Ty21a; unknown; Vi^−^ GalE^−^	13
χ8444	Ty800; Δ*phoPQ23*	14
χ9639	ΔP_crp527_::TT *araC* P_BAD_ *crp* ΔP_fur81_::TT *araC* P_BAD_ *fur* Δ*pmi-2426* Δ(*gmd-fcl*)*-26* Δ*relA198*::*araC* P_BAD_ *lacI* TT Δ*araE25* Δ*tviABCDE10* Δ*agfBAC811* Δ*sopB1925* Δ*asdA33*	16
χ9640	ΔP_crp527_::TT *araC* P_BAD_ *crp* ΔP_fur81_::TT *araC* P_BAD_ *fur* Δ*pmi-2426* Δ(*gmd-fcl*)*-26* Δ*relA198*::*araC* P_BAD_ *lacI* TT Δ*araE25* Δ*tviABCDE10* Δ*agfBAC811* Δ*sopB1925* Δ*asdA33* RpoS^+^	16
χ11498	Unknown; Vi^−^ GalE^−^ RpoS^*+*^	χ8205
χ11499	Δ*phoPQ23* RpoS^*+*^	χ8444
χ11513	Δ*aroC1019* Δ*aroD1013* Δ*htrA* RpoS^*+*^	χ8073
Plasmids		
pYA3467	Suicide vector containing *rpoS* from serotype Typhi ISP1820 used in generating RpoS^+^ strains	42
pYA3493	pYA3342 derivative β-lactamase signal sequence-based periplasmic secretion plasmid	7

## RESULTS

### RASTyV strains containing a functional RpoS are better able to survive when exposed to an acidic pH.

**FIG 1:**
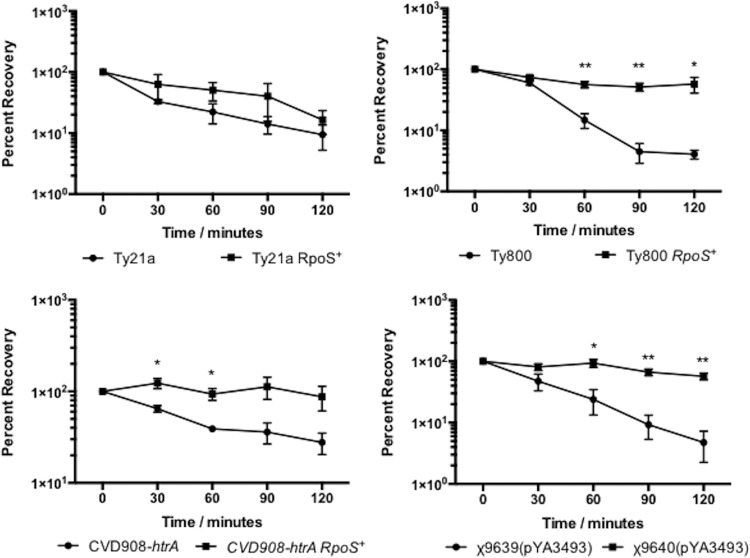
Acid tolerance response of RpoS^+^ RASTyV strains compared to RpoS^−^ strains at pH 3. Strains were grown overnight in EGA medium before being normalized to the same OD_600_ and then harvested by centrifugation and resuspended in EGA medium at pH 3. Cell viability was monitored by plating on LB agar containing all necessary supplements. The data presented are results from three independent experiments, and error bars are shown as standard errors of the means. *, *P* < 0.05; **, *P* < 0.01 using Student’s *t* test.

**FIG 2:**
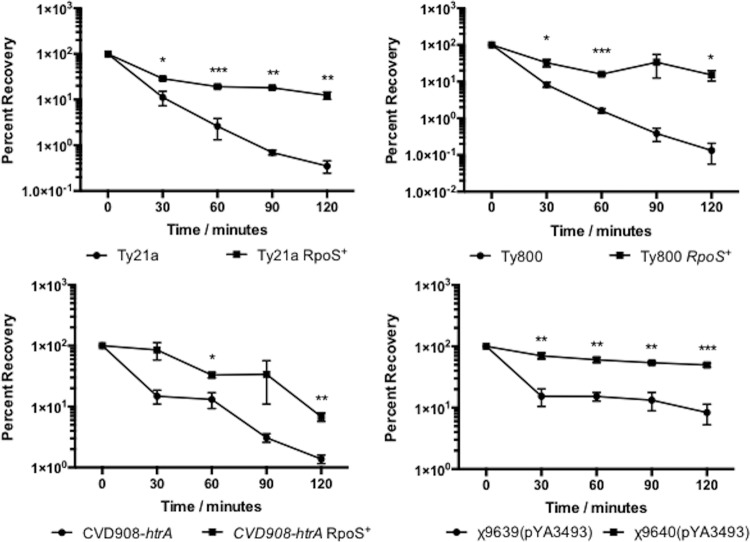
Acid tolerance response of RpoS^+^ RASTyV strains compared to RpoS^−^ strains at pH 2.5. Strains were grown overnight in EGA medium before being normalized to the same OD_600_ and then harvested by centrifugation and resuspended in EGA medium at pH 2.5. Cell viability was monitored by plating on LB agar containing all necessary supplements. The data presented are results of three independent experiments, and error bars are shown as standard errors of the means. *, *P* < 0.05; **, *P* < 0.01; ***, *P* < 0.001 using Student’s *t* test.

In order for orally ingested RASTyV strains to reach their target cells, they must pass through the hostile environment of the human stomach. In order to pass through this hostile environment, strains must be able to withstand the highly acidic pH of the human stomach, which is around 2.0 following a fast (40). During stationary phase, RpoS is an important regulator of the response to acid stress (33, 41). Therefore, we wanted to evaluate the ability of RpoS-positive vaccine strains to survive acidic environments compared to the RpoS-negative parental strains. To test this notion, strains were grown aerobically in minimal EGA medium to the stationary phase at pH 7 and then challenged at either pH 3 or 2.5 (Fig. 1 and 2). Under these conditions, we observed that at all times the RpoS^+^ strains were better able to survive than their parental RpoS^−^ strains, indicating that the presence of a functional RpoS greatly contributes to the ability of these RASTyV strains to survive upon exposure to acid stress.

### RASTyV strains containing a functional RpoS are better able to survive during starvation.

**FIG 3:**
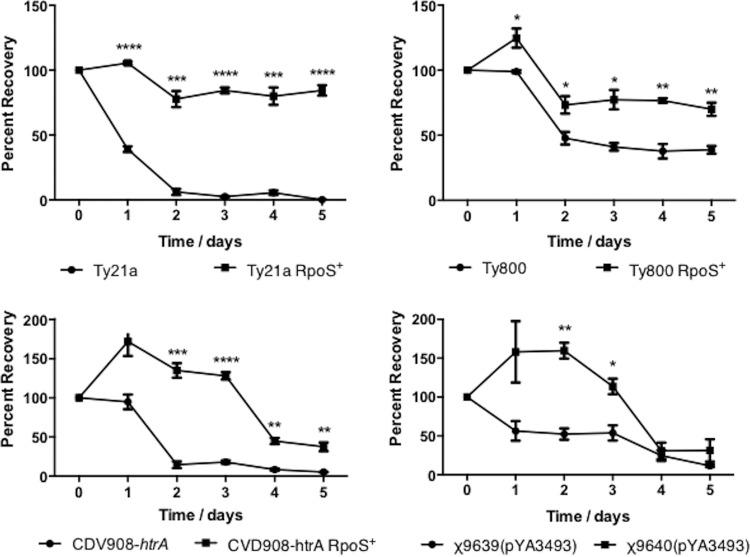
Growth of RpoS^+^ RASTyV strains in minimal medium compared to RpoS^−^ strains. Strains were grown overnight in LB medium before being normalized to the same OD_600_ and then harvested by centrifugation and resuspended in EGA medium. Cell viability was monitored by plating on LB agar containing all necessary supplements. The data presented are results of three independent experiments, and error bars are shown as standard errors of the means. *, *P* < 0.05; **, *P* < 0.01; ***, *P* < 0.001; ****, *P* < 0.0001 using Student’s *t* test.

The environment inside the human host is very nutrient limiting, and several studies have shown that RpoS plays an integral part in *Salmonella*’s response to nutrient deprivation (42, 43). Therefore, vaccine strains that contain a functional RpoS should be able to better cope with the nutrient-limiting environment of the host than strains that do not have this protein. To test this hypothesis, we grew strains overnight in LB broth that had been appropriately supplemented before normalizing the cultures to the same optical density at 600 nm (OD_600_) and then harvested cells by centrifugation. Following this, bacterial samples were washed and resuspended in Q3 medium, and cell viability was monitored every day for 5 days. The viability of the cultures during starvation was assessed by serial dilution and plating for CFU and comparing them to samples taken on day 0 (Fig. 3). For each set of strains evaluated, we observed that there was an increased survival of strains that contained a functional RpoS, indicating that these strains are more adept at surviving in nutrient-depleted environments such as those in humans.

### RpoS^+^ RASTyV strains are better able to survive during extended stationary phase.

**FIG 4:**
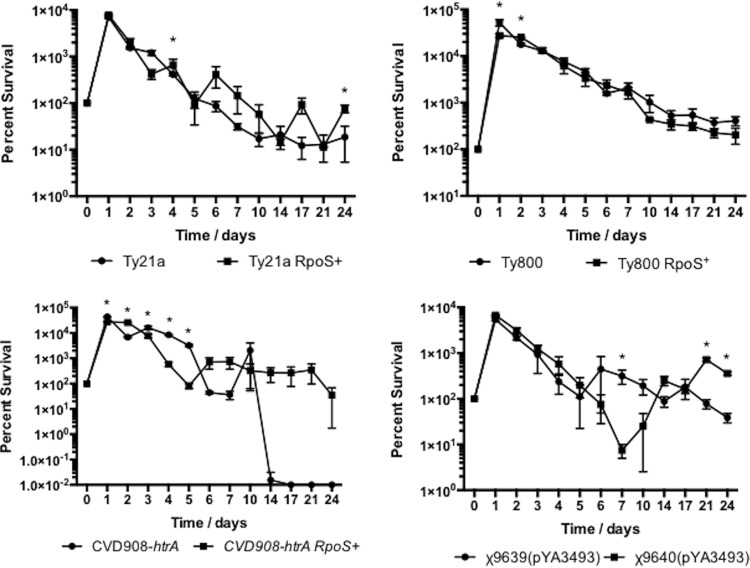
Evaluation of RpoS^+^ and RpoS^−^ RASTyV strains during survival in extended stationary phase. Strains were grown overnight in LB medium that was appropriately supplemented before being subcultured into fresh medium and allowed to grow for 24 consecutive days. Cell viability was monitored by serial dilution and plating on LB agar with supplementation. The data presented are results of 3 independent experiments, and error bars are shown as standard errors of the means. *, *P* < 0.05.

One of the major roles for RpoS is controlling the expression of genes that facilitate survival of the cell during the transition into stationary phase (30). Thus, it would stand to reason that RpoS^+^ RASTyV strains would be better able to survive than RpoS^−^ RASTyV strains during times of extended starvation, such as those encountered during stationary phase. Therefore, we wanted to evaluate the ability of the RASTyV strains containing an intact *rpoS* to survive during extended stationary growth compared to those strains that did not contain an intact *rpoS* (Fig. 4). We monitored the survival of all strains in LB medium, supplemented where appropriate, for a period of 24 days. We observed that for all strains, except for Ty800 and Ty800 RpoS^+^, survival rates were similar during the early part of the experiment, but during the last 7 days of the experiment, we began to recover more of the RpoS^+^ strains than the RpoS^−^ strains. However, for strains Ty800 and Ty800 RpoS^+^, we observed similar recoveries of viable cells for the two strains at all times. Taken together, these results indicate that the presence of a functioning RpoS protein contributes to the overall fitness of the vaccine strains compared to their RpoS-deficient counterparts.

### Effect of *rpoS* on Vi synthesis.

Previous studies have demonstrated that expression of the Vi antigen is influenced by the presence of RpoS (44). We hypothesized that the vaccine strains that contain a functioning RpoS express less Vi antigen, which may give them an advantage over the RpoS^−^ strain because they are less immunosuppressive. Because Ty21a and χ9639(pYA3493) contain a *tvi* mutation, rendering them negative for Vi synthesis, we focused on strains Ty800 and CVD908-*htrA* for this experiment (Table 2).

**TABLE 2:** O_9_ and Vi slide agglutination reactions of serotype Typhi strains

Strain	Genotype	Reaction for indicated antigen^a^	
		O_9_	Vi
χ3769	Ty2; RpoS^−^	−	+++
χ8073	CVD908-*htrA*; Δ*aroC1019* Δ*aroD1013* Δ*htrA* RpoS^−^	−	+++
χ8438	Ty2; RpoS^+^	+++	−
χ8444	Ty800; Δ*phoPQ23* RpoS^−^	−	+++
χ9197	Ty2; *ΔtviABCDE10* Cys^−^ Vi^−^ RpoS^−^	−	+++
χ9198	ISP1820; *ΔtviABCDE10* Cys^−^ Trp^−^ Vi^−^ RpoS^−^	+++	−
χ11499	Ty800; Δ*phoPQ23* RpoS^*+*^	+++	−
χ11513	CVD908-*htrA*; Δ*aroC1019* Δ*aroD1013* Δ*htrA* RpoS^*+*^	+++	−

a The degrees of agglutination ranged from not detectable (−) to strong (+++).

We observed that the RpoS^+^ strains had less agglutination when exposed to the Vi antiserum, indicating that these strains expressed lower levels of the Vi antigen, which corroborates previous studies that demonstrated that RpoS represses Vi synthesis (44). In addition, we evaluated agglutination against the lipopolysaccharide (LPS), or O antigen, because the O antigen is masked by expression of the Vi antigen (44). If lower levels of Vi are expressed, there will be more O antigen exposed, which will increase observed agglutination when strains are exposed to O-antigen antiserum. Therefore, in parallel with evaluating agglutination of the Vi antigen, we also evaluated the agglutination of our strains when exposed to O-antigen antiserum. We observed that in the parental RpoS^−^ strains there was less agglutination when they were exposed to the O antiserum due to expression of the Vi antigen, which masked the O antigen. However, we observed an increase in agglutination of the O antigen when the RpoS^+^ strains were exposed to O antiserum, in accord with a decrease in the production of the Vi antigen.

### RpoS^+^ *S*. Typhi strains are as resistant to guinea pig complement as RpoS^−^ strains.

**FIG 5:**
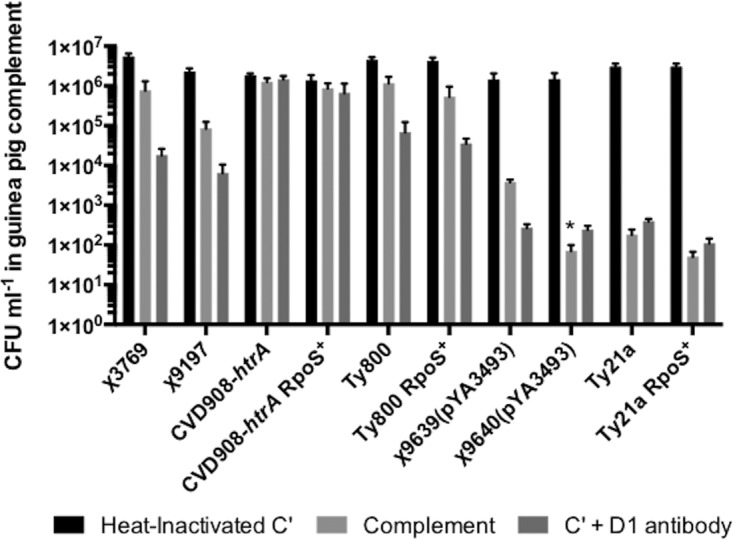
Evaluation of the survival of RASTyV strains in guinea pig complement. *S*. Typhi survival in guinea pig complement 3 h after inoculation with 1 × 10^6^ CFU. Data represented are the results of three independent experiments, and error bars are standard errors of the means. *, *P* < 0.05 using Student’s *t* test.

Complement is an important innate immune component of blood that enhances the abilities of antibodies and phagocytic cells to fight infection. We evaluated the sensitivities of the RpoS^+^ RASTyV strains in comparison with their RpoS^−^ parental counterparts to complement (Fig. 5). For strains Ty21a, Ty800, and CVD908-*htrA*, we did not observe any significant differences in the survival of the RpoS^+^ RASTyV compared to the RpoS^−^ strains in the presence or absence of anti-*Salmonella* O-antigen group D_1_ antibody. However, for strain χ9640(pYA3493), we observed a significant increase (*P* = 0.05 using Student’s *t* test) in sensitivity to complement in the absence of anti-*Salmonella* O-antigen group D_1_ antibody relative to its RpoS^−^ counterpart, χ9639. This increase in sensitivity was not observed in the presence of the anti-*Salmonella* O-antigen group D_1_ antibody. This is consistent with work previously performed in our lab that demonstrated that RpoS^+^ RASTyV vaccine strains are as safe as RpoS^−^ RASTyV strains (14).

### RASTyV strains containing a functional RpoS are better able to survive when exposed to hydrogen peroxide.

**FIG 6:**
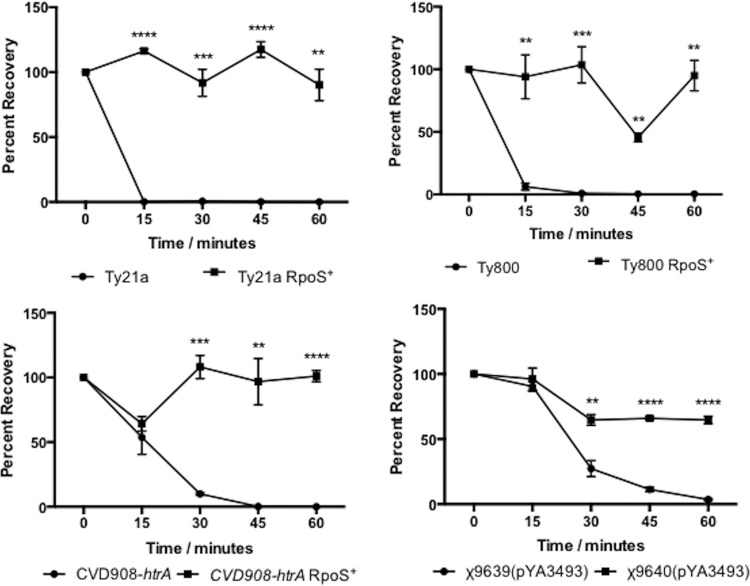
Evaluation of RpoS^+^ RASTyV strains when exposed to hydrogen peroxide compared to RpoS^−^ strains. Strains were grown overnight in LB medium before being normalized to the same OD_600_ and then harvested by centrifugation and resuspended in BSG and 19.58 mM hydrogen peroxide. Cell viability was monitored by plating on LB agar containing all necessary supplements. The data presented are results of three independent experiments, and error bars are shown as standard errors of the means. **, *P* < 0.01; ***, *P* < 0.001; ****, *P* < 0.0001 using Student’s *t* test.

It is well documented that RpoS plays a role in modulating the expression of genes that are required in the transition into stationary phase, including those that help combat oxidative stress. Macrophages of the human body engulf and kill bacteria through a number of mechanisms that include the use of reactive oxygen species (ROS) (45). We wanted to evaluate the ability of RASTyV strains containing a functional RpoS to survive exposure to hydrogen peroxide compared to those strains that do not have a functional RpoS. To accomplish this, we subjected stationary-phase cultures of each strain to 19.58 mM hydrogen peroxide and measured cell viability by serial dilution and plating on LB containing the appropriate supplements (Fig. 6). In each experiment, we observed a statistically significant increase in the survival of those strains that contained a functioning RpoS protein. These results suggest that RASTyV strains that contain a functioning RpoS would survive better inside the host than those strains that contained the mutant allele producing an altered RpoS.

## DISCUSSION

The use of live attenuated *Salmonella* strains in the prevention of infectious diseases is an attractive alternative to the use of subunit or killed vaccines. Indeed, there are several vaccines that utilize live attenuated *Salmonella* to vaccinate against typhoid, including Ty21a, CVD908-*htrA*, and Ty800 (15, 16, 19, 20). These vaccine strains are derived from the Ty2 strain of *S*. Typhi, which contains a mutation in the alternative sigma factor RpoS that renders it nonfunctional (28). Previous clinical trials performed by our lab indicate that RpoS^+^ *S*. Typhi vaccines are superior to the RpoS^−^ *S*. Typhi vaccines (14). Therefore, we postulated that RpoS^+^ derivatives of existing *S*. Typhi vaccine strains should be better able to withstand environmental stresses than the RpoS^−^ parent strains. In the present study, we describe the conversion of 4 clinically relevant typhoid vaccine strains derived from Ty2 [CVD908-*htrA*, Ty800, Ty21a, and χ9639(pYA3493)] to RpoS^+^ and evaluate their ability to survive under various stresses compared to their parent strains. If so, the RASTyV strains containing a functioning RpoS should be better vaccine candidates for further development as live attenuated recombination *Salmonella* vaccine strains.

RpoS has been extensively studied in Gram-negative bacteria such as *Escherichia coli* and has been shown to play an important role in the survival of cells upon exposure to acidic environments (32, 46). This type of environment is commonly encountered in the host, where the pH of the stomach is approximately 2 following a fast (40). The ability of an orally administered live attenuated strain to survive greatly adds to its attractiveness because it must pass through the stomach to reach the gut-associated lymphoid tissue (GALT) in the ileum where it will invade and lead to the induction of mucosal, systemic, and cellular immune responses (39). Therefore, we set out to determine how the newly constructed RpoS^+^ RASV strains compared to their RpoS^−^ parental counterparts. Following acid challenge, we observed that the RpoS^+^ strains were better able to survive than the RpoS^−^ strains. These results were not surprising given that this regulator is responsible for the transcription of genes involved in acid resistance, such *gadC*, a gene of the glutamate-dependent low-pH-resistance network of genes; *hdeAB*, which encodes pH-regulated periplasmic chaperones; and *cfa*, which encodes a protein for cyclopropane fatty acid synthesis (47, 48). Indeed, in a previous study with *S*. Typhimurium it was observed that strains lacking a functional RpoS were unable to survive following exposure to a pH of 3.0 (33, 49). Further to this, the transient exposure to the acidic environment of the stomach serves as a signal to *Salmonella* that it has entered the host and this allows the invading cells to prepare for the stresses that will be encountered in the intestine and in invaded tissues. The presence of a functional RpoS might eliminate the need for low-pH-bypass strategies (i.e., bicarbonate, encapsulation, etc.) that are often used in parallel with orally administered vaccine strains and enhance the immunogenicity of the vaccine strain through improving interaction of the strain with host tissue.

As with exposure to acid, nutrient deprivation acts as an important regulatory signal in the expression of genes involved in virulence in *Salmonella*. In addition, RpoS positively regulates a number of genes that are involved in *Salmonella*’s increased resistance to environmental stresses, including nutrient deprivation (50). Since the ability to overcome starvation is important in the ability to establish infection, we evaluated the ability of the RpoS^+^ strains to survive under nutrient-limiting conditions, i.e., growth in minimal medium. We expected to observe that the presence of RpoS would enhance the ability of our vaccine strains to survive when grown under nutrient deprivation conditions, which is what we observed. These results are expected given that previous research with *S*. Typhimurium demonstrated that not only is RpoS involved in the regulation of the starvation-inducible loci (*sti*) but also it is reportedly positively regulated by ppGpp during starvation (50).

The entrance into stationary phase is, in part, marked by the depletion of key nutrients and subsequently an elevation of RpoS. Therefore, in addition to evaluating starvation (simulated by growth in minimal medium), we evaluated the ability of the vaccine strains to survive during an extended stationary phase that was simulated through growth in rich medium for 24 days. While we recovered various numbers of CFU during the duration of the experiment, for strains CVD908-*htrA*, Ty21a, χ9639, and their RpoS^+^ counterparts, we did observe that the RpoS^+^ strains generally outcompeted their parent strains. Again, these results are not surprising because as cells enter stationary phase, the transcription and translation of RpoS are significantly increased (50). RpoS is either directly or indirectly responsible for controlling the expression of a large number of genes that are involved in helping *Salmonella* survive stationary phase (30, 50, 51). For Ty800 and its RpoS^+^ derivative, it appeared that the parental strain outcompeted the RpoS^+^ strain, although it was not significant. This may be due to the already-existing *phoPQ* mutation, which renders the strain devoid of sensing and responding to extracellular levels of magnesium through the PhoPQ two-component system. Magnesium is an essential nutrient in the physiology of all microorganisms and an important cofactor to a number of enzymes (52). It stands to reason that perhaps the RpoS^+^ strain does not outcompete the wild type because both strains possess the *phoPQ* mutation, which could lead to a decrease in intracellular levels of magnesium and the ability of these strains to survive during an extended stationary phase.

One mechanism by which *S*. Typhi evades the host immune response is through the synthesis of a capsule, also known as the Vi antigen (53). Indeed, it has previously been reported that the Vi antigen blocks the deposition of C3b on the surface of *S*. Typhi, thereby making it more resistant to complement killing via the alternative pathway and phagocytosis (53, 54). Further to this, a previous study demonstrated that synthesis of the Vi antigen is negatively controlled by the alternative sigma factor RpoS (44). While strains that express the Vi antigen are less adherent to and invasive into epithelial cells, this antigen is not expressed in high-osmolarity media, such as in the intestinal lumen; therefore, it would stand to reason that RpoS^+^ vaccine strains would be better able to survive following entrance into the host and allowed to reach the Peyer’s patches, where they will stimulate the mucosal immune system.

Complement is an important blood component that possesses antimicrobial effects. Resistance to complement-mediated killing is an important trait of Gram-negative bacteria, including *Salmonella*. In *Salmonella*, this is mainly due to the production of the Vi antigen, which has been shown to prevent deposition of C3b and the membrane attack complex (C5b-9) on the surface of *S*. Typhi, therefore resulting in decreased complement- and antibody-mediated killing (53, 54). Indeed, the ability of RASTyV strains to survive exposure to complement would be greatly advantageous as it is likely that these strains would remain viable long enough for the strains to reach deep effector lymphoid tissues. Previous studies have demonstrated that Ty21a and CVD908-*htrA* are adept at mimicking natural infection in stimulating both mucosal and systemic immune responses, thus leading to the conclusion that these strains are able to survive exposure to complement (16, 55). Since previous studies have demonstrated that RpoS negatively affects the production of the Vi antigen, we aimed at evaluating if introducing a functioning RpoS into these strains would negatively affect their ability to resist complement-mediated killing (44). The results of this study indicate that the RpoS^+^ strains were no more resistant than their RpoS^−^ parental strains.

The ability to overcome exposure to reactive oxygen species (ROS), such as hydrogen peroxide, would greatly enhance the delivery of RASTyV strains to deep effector lymphoid tissues. The ability of *Salmonella* to invade and survive within phagocytic cells is an important part of its life cycle within the host (56). Therefore, this organism has evolved a number of mechanisms that overcome exposure to dangerous ROS, which are a critical component of the host’s antimicrobial arsenal (35, 36, 56–58, 66). In the present study, we demonstrated that the RpoS^+^ RASTyV strains were better able to withstand exposure to lethal concentrations of hydrogen peroxide than the RpoS^−^ counterparts. It is well known that in *S*. Typhimurium and *E. coli*, RpoS controls the expression of *katE*, which encodes hydroperoxidase II that is responsible for the deprotonation of hydrogen peroxide to water and oxygen under nutrient-limiting conditions (57, 59). In addition, RpoS controls the expression of *dps*, which encodes DNA protection during starvation (60). Upon entrance into stationary phase, Dps becomes the most abundant protein in the cytoplasm (61). Dps is responsible for binding nonspecifically to DNA and forming a crystalline structure that compacts the DNA, thereby protecting it from oxidative damage (61). The ability to combat oxidative stress through the expression of RpoS-regulated genes would be beneficial to RASTyV strains because an important issue in developing live attenuated vaccine strains is their ability to invade and colonize deep effector lymphoid tissues following mucosal delivery.

In conclusion, the results of this present study demonstrate that RpoS^+^ variants of Ty21a, Ty800, CVD908-*htrA*, and χ9639(pYA3493) are better able to cope with a variety of environmental stresses likely encountered in the human host after oral administration. Since *S*. Typhi is human host specific and is unable to infect mouse tissues, we undertook *in vitro* studies to simulate conditions likely to be encountered in the orally vaccinated human host. Our studies indicate that RpoS^+^ RASTyV strains would be better able to combat the harsh acidic domain of the human stomach than the RpoS^−^ strains. In addition, our studies indicate that RpoS^+^ RASTyV strains would be better able to survive nutrient-limiting conditions when grown in both minimal and rich culture media, suggesting that these RpoS^+^ strains would be better at adapting to the nutrient-depleted environment of the host. These studies demonstrated that the RpoS^+^ strains would be better able to survive the onslaught brought on by various host defense and innate immune responses, as demonstrated by these strains’ ability to survive exposure to lethal levels of hydrogen peroxide. Further to this, the RpoS^+^ RASTyV strains are not rendered more sensitive to complement-mediated killing than their RpoS^−^ counterparts. These findings further support the development and use of the newly constructed RpoS^+^ RASTyV strains (which we will make freely available upon request) as safe, effective vaccines to prevent *S*. Typhi infections or as vectors in recombinant attenuated *Salmonella* vaccines designed to protect against other infectious disease agents.

## MATERIALS AND METHODS

### Bacterial strains and growth conditions.

*Escherichia coli* and *S. enterica* serovar Typhi strains, along with plasmids and primers, used in this study are listed in Table 1. Cultures were routinely grown on LB plates or in LB broth at 37°C. For acid shock and starvation assays, cultures were grown in minimal E medium at pH 7 supplemented with 0.4% glucose (EG medium [62]). When indicated, medium was further supplemented with 25 μg ml^−1^ chloramphenicol (Cm), 0.2% (wt/vol) arabinose, 0.2% (wt/vol) mannose, 50 μg ml^−1^ 2,6-diaminopimelic acid (DAP), 22 μg ml^−1^ l-cysteine–HCl, 20 μg ml^−1^ l-tryptophan, 0.1% Casamino Acids, 2 μg ml^−1^ *p*-aminobenzoic acid (PABA), and 2.5 μg ml^−1^ 2,3-dihydroxybenzoate (DHB).

### Construction of RpoS^+^ strains.

The RpoS^+^ strains were constructed using the recombinant suicide vector pYA3467 (9.6 kb), carrying the *rpoS* gene from serotype Typhi ISP1820 (993 bp) between flanking regions (338 bp 5′ and 83 bp 3′) of the *rpoS* allele in serotype Typhi Ty2, as previously described (44). The presence of the RpoS^+^ insertion in χ9640(pYA3493), χ11498 (Ty21a RpoS^+^), χ11499 (Ty800 RpoS^+^), and χ11513 (CVD908-*htrA* RpoS^+^) was confirmed by a catalase activity assay (44).

### Acid shock assays.

Overnight cultures of Ty21a, Ty800, Ty21a RpoS^+^, and Ty800 RpoS^+^ were set up in EG medium (pH 7) supplemented with l-cysteine–HCl, tryptophan, and Casamino Acids (EGA medium), while CVD908-*htrA* and CVD908-*htrA* RpoS^+^ were set up in EGA medium supplemented with 2 μg ml^−1^ PABA and 2.5 μg ml^−1^ DHB and χ9639(pYA3493) and χ9640(pYA3493) were supplemented with 50 μg ml^−1^ DAP and 0.2% arabinose. Cultures were normalized to the same optical density (OD_600_) before being harvested by centrifugation and washed with EGA medium (pH 7.0). The cells were then pelleted and resuspended in either EGA medium or EGA medium that was supplemented with either DHB and PABA or arabinose and DAP at pH 3 or 2.5. Cultures were placed at 37°C, and samples were collected every 30 min up to 120 min. Samples were then serially diluted, and CFU per milliliter were determined alongside control samples that were removed immediately following resuspension in acidified EGA medium. Percent recovery was calculated by comparing the initial CFU per milliliter to final CFU per milliliter from three independent experiments.

### Starvation assay.

All strains were grown overnight in LB broth or LB broth that had been supplemented. Overnight cultures were diluted 1:100 in fresh medium and grown to an OD_600_ of 0.3 to 0.4 (exponential phase). One milliliter of this culture was then harvested via centrifugation and washed once in buffered saline gelatin (BSG) before being resuspended in Q3 medium [60.28 mM K_2_HPO_4_, 33.07 mM KH_2_PO_4_, 14.67 mM (NH_4_)_2_SO_4_, 3.4 mM Na_3_C_6_H_5_O_7_, 0.02% MgSO_4_, 0.004% histidine HCl, 0.0005% thiamine HCl, and 1% glucose] supplemented with 0.1% Casamino Acids, 22 μg ml^−1^ cysteine, 20 μg ml^−1^ tryptophan, 50 μg ml^−1^ phenylalanine, and 20 μg ml^−1^ tyrosine. In addition, medium for strains CVD908-*htrA* and CVD908-*htrA* RpoS^+^ was further supplemented with 2 μg ml^−1^ PABA and 2.5 μg ml^−1^ DHB and medium for strains χ9639(pYA3493) and χ9640(pYA3493) was additionally supplemented with 0.2% arabinose. The cultures were then incubated at 37°C with aeration for 5 days. At the desired times, samples were taken, serially diluted in BSG, plated onto LB agar or LB agar that had been supplemented, and placed at 37°C to determine viable counts. Percent survival was calculated by comparing the initial CFU per milliliter to final CFU per milliliter from three independent experiments.

### Survival during extended stationary phase.

Long-term stationary-phase survival was measured as previously described (63). Briefly, overnight cultures were grown in LB broth or LB broth that had been supplemented with DHB and PABA or arabinose and DAP at 37°C with aeration. At this point, aliquots were removed for day 0 viable counts. The cultures were then maintained in the original culture medium for an additional 24 days with aeration at 37°C, and aliquots were removed at the specified times. The samples were serially diluted in BSG and plated onto LB agar or LB agar that had been supplemented. Percent recovery was calculated by comparing the initial day 0 CFU per milliliter to final CFU per milliliter from three independent experiments. Statistical significance was determined using a Student’s *t* test with a 5% confidence interval.

### Agglutination assays.

Agglutination tests were performed as previously described (44). Briefly, agglutination tests were performed on glass microscope slides by mixing 50 μl of antisera against Vi and O_9_ (Difco Laboratories, Detroit, MI) with suspensions of single colonies. The reactions were visualized by phase-contrast microscopy at 10× magnification.

### Complement resistance assay.

The complement resistance assay was performed as previously described (64). Briefly, cultures of Ty21a, Ty21a RpoS^+^, Ty800, Ty800 RpoS^+^, CVD908-*htrA*, CVD908-*htrA* RpoS^+^, **χ**9639(pYA3493), and **χ**9640(pYA3493) were grown in LB broth overnight at 70 rpm at 37°C. The following day, the overnight cultures were subcultured into fresh medium and allowed to grow to an OD_600_ of 2.0 to 2.1, which corresponds to a cell density of approximately 1.0 × 10^9^ CFU ml^−1^. Cells were then diluted to a density of 1.0 × 10^6^ CFU ml^−1^ in phosphate-buffered saline (PBS) and then exposed to 22% purified guinea pig complement (Calbiochem, San Diego, CA) in the presence or absence of group D_1_ lipopolysaccharide (LPS) O-antigen antibody (BD Bioscience, Franklin Lakes, NJ). Reaction mixtures were incubated for 3 h at 37°C. Complement-resistant cells were then enumerated by plating on LB agar or LB agar that was supplemented. The assay was conducted in duplicate and was repeated a minimum of 3 times for each strain.

### Sensitivity to hydrogen peroxide.

Sensitivity to hydrogen peroxide was determined as previously described (65). Briefly, exponentially growing cultures were washed and resuspended in phosphate-buffered saline (PBS) before the addition of 19.58 mM H_2_O_2_. Cultures were placed at 37°C with shaking, and aliquots were removed at the time intervals specified. Samples were then serially diluted, and CFU per milliliter were determined alongside control samples that were removed prior to the addition of H_2_O_2_. Percent survival was calculated by comparing the initial CFU per milliliter to final CFU per milliliter from three independent experiments.

### Statistical analysis.

Numerical data are presented as arithmetic means for bacterial number data. Statistical significance was determined using Student’s *t* test with a 5% confidence interval.
